# Provings and Clinical Observations with High Potencies

**Published:** 1892-03

**Authors:** Malcolm Macfarlan

**Affiliations:** Philadelphia


### PROVINGS AND CLINICAL OBSERVATIONS WITH HIGH POTENCIES.

MALCOLM MACFARLAN, M. D., PHILADELPHIA.

(Continued from February number, page 60.)

### PHOSPHORUS$^{CM}$.

Cured scintillations of light before the eyes. Cured some cases of weakness of sight (asthenopia). The prover is nervous, excitable, tremulous, has pain in theright side of the small of the back, *cramps in the stomach*, vivid dreams, burning pain in the small of the back, burning pain and cramps in stomach, vertigo and feeling of emptiness in the head. Mental effort causes fatigue. The *burning in the stomach* extends up to the œsophagus, verified this often ; a feeling of constriction ; dryness of the fauces, bloated stomach, and abdomen.

Choking, disposition to swallow constantly. Pain comes down line of sternum to epigastrium.

### PHYSOSTIGMA–VEN.$^{CM}$.

Severe cramps in stomach, disposition to vomit, nausea, bowels very loose and light in color, watery stools with cramps. Attacks of trembling. Profuse perspiration, lasting a short time.

### PHYTOLACCA–DECAN$^{47M}$.

Tight, dry cough, attended with pain from middle of sternum through to the back. Cough aggravated by lying down.

Swelling on and around the left ear and side of the face, like erysipelas ; scalp sore, painful to touch. Chilly every morning ; sleepy ; bowels costive ; skin dry ; pain across kidneys ; urine red and muddy. Joints of lower extremities ache. Dull pains across forehead ; feels wretched on getting up ; ache from shoulder to hip ; sharp pains shooting through the ball of the eye on reading or writing ; poor rest ; appetite good. Painful red points on the tongue ; great pain down the sides of the hips and thighs, knee and ankle joints. Pain in lower extremities causes her to cry out. It is constant in the right elbow joint, at intervals down the muscles of the forearm.

### PIMPINELLA-SAX.$^{10M}$.

Gurgling, frequent rumbling noise in abdomen; no pain; seems like formation of an undue amount of gas as in dyspeptic cases.

### PIX-LIQUIDA$^{5C}$.

Expectoration constant and profuse, occasionally mixed with blood; gums sore and teeth sensitive; headache and sore scalp, left side of head more affected than the right; dryness and burning at root of nose, burning and tingling about nose. Cough and irritation of skin with disposition to scratch in all provers.

### PLANTAGO-MAJ.$^{5C}$.

Profuse sweat toward evening, followed by sleepy feeling. Awoke in a fright; passes mucus from bowels, which are disposed to be loose; rumbling in abdomen, flatulency; general weakness; seems as if everything was a burden.

### PLANTAGO-MINOR$^{11C}$.

Burning pain across the buttocks; great soreness and throbbing on top of the head, sensitive to touch; eyes are weak, not inflamed, vision blurred; bowels constipated; piles. Stiff neck, hardly able to turn it; pain in the lower part of abdomen; *soreness in wrist joint* (mostly right); little finger of right hand stiff; dry tongue; short breathed; pain in the back near waist; scalding sensation in passing water; stiff wrist and ends of fingers affected as if sore or sensitive. Symptoms developed on second day. She thinks she would choke and perhaps die, because of dryness and constriction in throat. Spasms of muscles of extremities; suddenly breaks out in great heats; startled as if frightened for a moment or two; teeth become set or closed and she cannot open her mouth for a time; pains through her teeth; felt as if she needed air. Choking sensation. Swallowing is difficult, as if there were no power to force down the bolus.

Sudden sharp pain through her head just above the ears, great soreness. Urine very dark in color.

Woman, given in water for three days, every two hours. "Suffering terribly in her right lower limb from the knees to the hip joint and then around to the back, not constant pain, but it comes quick as lightning with a zig-zag course, sometimes every few seconds and then perhaps after twenty to thirty minutes. She screams out. It leaves a soreness of the muscles." A physician called in temporarily during her suffering diagnosed sciatica.

Woman aged forty. Eyes affected in a way she can't explain, simply does not wish to use them. Urine appears like coffee. Taking medicine every half hour during the day for a fortnight. Seized during night with severe pain, like rheumatism; couldn't bear to touch the middle of arm, it is so sensitive; pain in forehead; heat in hands and head; twisting pain in abdomen with a desire to move the bowels, she tries but accomplishes little. Piles bad, she can scarcely endure them, not subject to this. Her eyes are dim, as if looking through smoke. Watering of eyes. Symptoms are given in the language of the provers to convey, if possible, an idea of the spirit of the provings.

### PLUMB-ACET.$^{1M}$ (F.).

Caused very free, watery movements from the bowels six to eight times a day. I have found this remedy useful in constipation, when the stools were hard, dry, and difficult of expulsion and light in color.

### POLYGONUM-HYDROP.$^{45M}$.

Very sick at stomach; *severe aching from her knees to her feet;* restless in bed from pain in extremities; wakefulness; no appetite; aching in legs, and sensitiveness. Face broken out in pustules; bowels, which were constipated, move regularly; menses (had amenorrhœa) returned, but were scanty, and very dark; limbs ache, and chilly, followed by fever in the afternoon.

### PSORIN$^{42M}$.

Caused eczema, with disposition to crusts behind the ears; bowels always disposed to be loose, now costive, and cured completely a young child's chronic deafness of a mild type. *Eyes*

*watered* and inflamed. Her eyes pained so she could scarcely open them. Sensitive over the eyebrows, down the nose, and back of the head. She complained mostly of her head.

A young child who was *always* pale, sickly, and delicate, and who every few days was disposed to very loose bowels was cured of its diarrhœa in a short time by Psorin. Since then it has gained greatly in weight. Have often verified this. It is curative in the diarrhœa of scrofulous children.

Appetite much better. She must eat in middle of the night. Sharp pain at the right side, opposite tenth rib. More extended provings brought out following observations:

Caused the nose to discharge freely, and cured a child's purulent otorrhœa. The latter symptom verified often.

Caused soreness below middle of sternum on pressure; a great deal of flatulency; breathing oppressed; distress in epigastrium; bowels, that were regular, now constipated, frequently verified. Eyes and ears, symptoms as above. Slight burning on passing water. Pain through right groin. Her eczema much better. Lids of left eye greatly inflamed; lachrymation.

Bowels loose; move four or five times a day; nervous; easily startled; pains across the epigastrium, and remain in the spleen, as soreness; severe pain in the head; pain in his ear so great as to confine him to bed four days; auricle much swollen.

Cured several chronic cases of otorrhœa in adults.

Soreness in jaw, right side, around the ear; could not open his mouth without great pain; could scarcely crowd his fingers between his teeth; tear-sac inflamed; epiphora.

### PULSATILLA$^{CM}$.

Soreness within the chest at site of both nipples; giddiness; increase of mucus from throat.

Very efficacious in curing old, fistulous openings in lachrymal sac; thick and yellow discharge. Chronic cases; many verifications.

Pain in his right groin, sharp; would come and go; was very severe; slept none for two nights with it; tears would run down his cheeks because of it. When he eats or drinks anything, it causes *cramps* in pit of stomach ; thirst for liquids of all kinds; crampy pains in abdomen, low down.

### RANUNCULUS-ACRIS $^{10M}$.

Stopped up in nose, burning in hands and face.

Eyes very weak, and water very much.

10M. Eyes very itchy ; light affects them much ; roof of her mouth sore ; sensation of lump in her throat ; difficult to swallow, as if tonsils were enlarged ; legs hurt her very much from knees downward as if they were rheumatic ; urine increased in quantity ; disposed to pass it often, with symptoms of scalding along urethra.

The urinary symptoms are the most prominent and constant.

### RHODODENDRON $^{CM}$.

*Toothache ;* facial neuralgia ; all the teeth get loose ; snags come away ; gums much swollen.

Astonishing cure of neuralgia ; inferior and superior dental nerves ; existing seven weeks ; woman in agony ; sleepless ; first dose relieved her ; been well ever since ; gums swollen ; previous to this was under old-school treatment and had three sound molars removed without relief.

Stiff neck ; gums and teeth sore ; pains various parts of body.

Frequently cured facial neuralgia with sensitive teeth.

### RUMEX-CRISP. $^{45M}$.

Caused a constant dry cough to be loose, and although chronic or lasting many months, and the woman disposed to consumption, it was greatly relieved in a week.

Aching pain from the shoulder-blades all the way down the back to region of the kidneys ; helps the cough of consumptive persons wonderfully ; repeatedly verified.

### RHUS-TOX. $^{105\,M}$.

Gives great relief, and often curative in ciliary neuralgia, with severe pains through the eye, and in cheek-bones down the side of the nose over the eye, with intolerance of light.

Abdomen sore below the navel; sensitive calves; inflamed throat; tonsillitis; mouth sore; conjunctivitis; rheumatic pains in muscles and joints; intolerable and uncontrollable itching of the feet and legs (worse at night); constantly rubbing one foot against the other, so itchy; sleeplessness because of itching skin; skin covered with a red, uniform, solid rash; slight conjunctivitis. Rash developed in majority of provers.

Cured many cases of *scald head* in children. The eruption covering the scalp and extending to the surrounding skin in some cases; curative in some cases of slight discharge from ears.

Cured permanently many persons who had always been subject to muscular rheumatism.

Removed chilblains; often verified.

Not much use in ophthalmia where there is photophobia, but *vice versa*.

This remedy, under certain conditions, must be the similar in hives, as well as in scarlet fever.

### SACCH-LACT.$^{CM}$.

In the early part of proving, feels very badly; unfit for work; weak.

Caused dizziness, nausea, and occasional vomiting; after taking the medicine for a week urine became scant and dark; have frequently cured vertigo, nausea, and nervousness with this remedy; head symptoms somewhat resemble Belladonna.

### SAP-SODA$^{2C}$.

Soda soap 2C. Dull, heavy pain across the eyes, and forehead; roughness in the skin; dry tetter; face looks swollen; with purple spots. I think the medicine produced a *boil* on a prover's left shoulder after taking it for a week.

Cold in her chest; all her joints ache; bottom of both heels ache; right one worse; feeling of tightness at middle of sternum; stiff and aching all over from head to foot; abdomen and chest feel sore and bruised; coughing painful; coughs a good deal; eyes very weak; reading is difficult; cured prover of dyspepsia, and bowels now constipated that were regular.

2C. Great soreness in her right kidney; severe pain extending down through her right groin and prevents her walking.

SALVIA-OFF.[5C].

*Giddiness;* sharp pains at the apex of the right lung, going through to the back; feels nauseated in the morning; *pains around her waist* and stomach; five free movements from her bowels from seven to nine o'clock in morning, then no more; *gnawing at pit of stomach;* the sensation rises upward toward the throat, and she is forced to take a deep breath to be relieved.

SAMBUCUS-NIGRA.[45M.]

Great swelling; heat and redness in glands of the neck accompanied by soreness; improved defective hearing; restless at night.

Symptoms of a common cold; eyes inflamed; rapid pulse; dry, hot skin; *complete loss of appetite;* general bruised feeling; tongue sore, and very much coated.

*Feeling of great general soreness, as if beaten;* dull, frontal headache; high fever; *erysipelas-like redness over the whole left side of the head; swelling of the ear;* confined to the bed; vivid, frightening dreams; great soreness in the abdomen; bowels move twice daily; feels very tired; after slight exertion legs feel very weak; thirsty; pain in his abdomen keeps him awake; eating causes nausea.

SANGUINARIA.[24M].

Soreness down the muscles of his back, on either side of spine; feels it more on breathing deeply; pain shifts about; sores around the margins of the gums; sores in roof of mouth.

Feels suddenly warm; only lasts a few minutes; sharp, frontal headache. Did not make extended provings of this remedy.

SANTONINE, 10TH DECIMAL TRITURATION.

Has cured very many cases of scrofulous ophthalmia and pannus in children; corneitis; great intolerance of light; constant watering of eyes; relieved adults almost as quickly; children cured usually had sore nostrils. Cured several cases of chronic otorrhœa with fistulous opening in mastoid cells.

Caused coryza; sneezing at all times, day and night; have usually followed this medicine with Sulphur.

Highly curative in nasal catarrh, of children particularly.

### SARRACENIA-PURP.$^{2M}$.

Sharp pain through the right lung to his shoulder-blade, commencing three inches below right nipple, and running directly backward to scapula.

Light-headed; weak; weakness between the shoulders, and below them; soreness in umbilicus; hungry all the time, even after meals.

### SASSAFRAS$^{5C}$.

Toothache; pain like a coal of fire on passing water; urine looks as if it had flakes of mucus in it; pain in both tubers ischii, and intense pain in both hip-joints; abdomen appears distended, and considerable eructation.

